# Supplementary material for: Climate-forced Hg-remobilization associated with fern mutagenesis in the aftermath of the end-Triassic extinction
Source: Nat Commun. 2024 Apr 27;15:3596. doi: 10.1038/s41467-024-47922-0 (PMC11519498; doi:10.1038/s41467-024-47922-0)
Supplement: Supplementary file 1 — Supplementary Information [file 41467_2024_47922_MOESM1_ESM.pdf]

Supplementary Files

Supplementary Information for "*Climate-forced Hg-remobilization associated with fern mutagenesis in the aftermath of the end-Triassic extinction*" by Bos *et al*.

Content:

Supplementary Fig. 1: Spore teratology images from Schandelah-1 core

Supplementary Fig. 2: Mercury-organic carbon/matter relations for Schandelah-1 (Lower Saxony Basin)

Supplementary Fig. 3: Mercury concentration and isotope relation to vegetation dynamics

Supplementary Fig. 4: Correlation of δ^{13}Corg records, bulk sedimentary Hg and Hg/TOC concentrations

Supplementary References

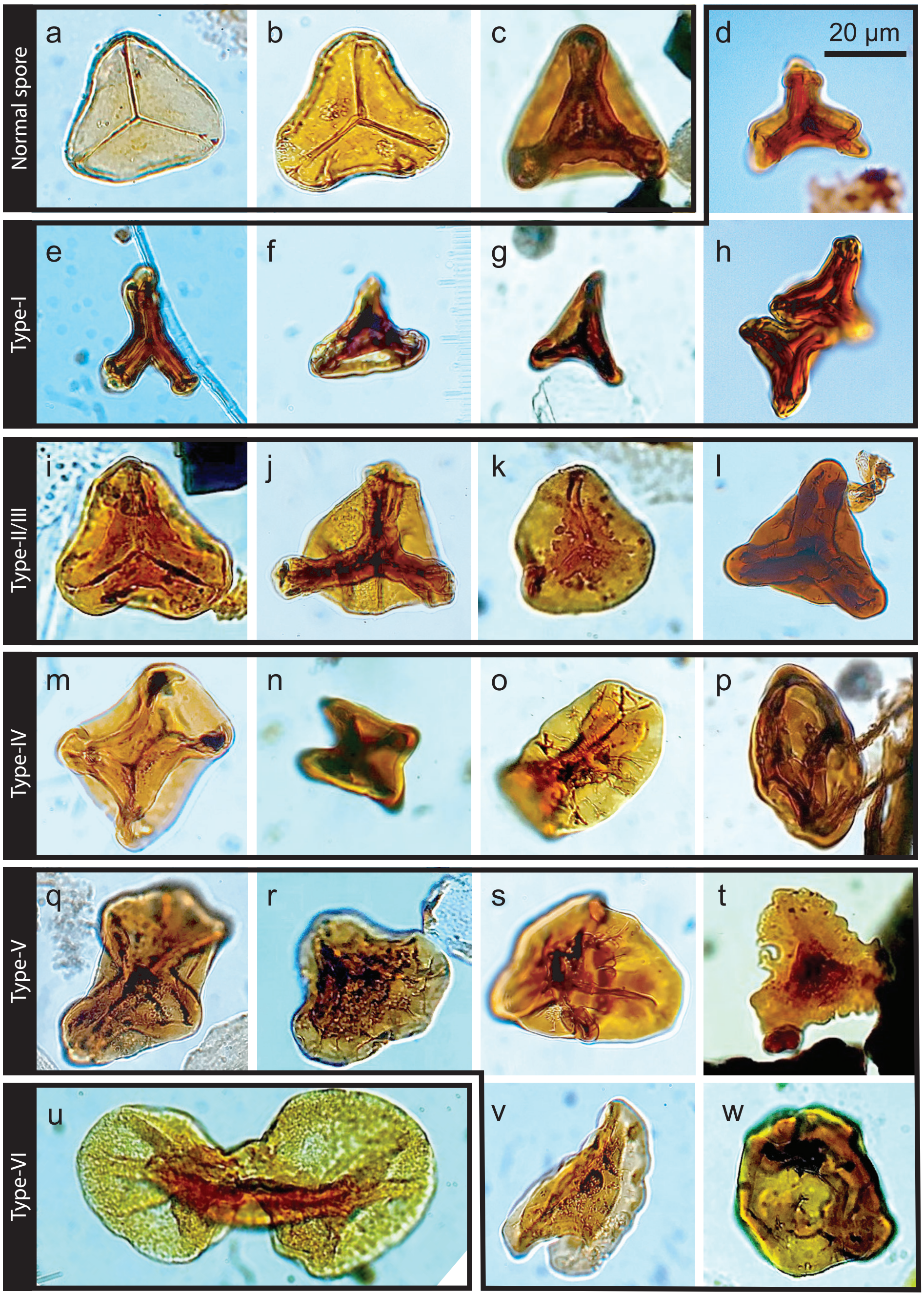

Supplementary Fig. 1. Spore teratology images from Schandelah-1 core. Normal spores: representatives of normal (**a** and **b**) Deltoidospora spp. specimens and (**c**) Concavisporites spp. specimen. Type-1 malformations: (**d** and **e**) unexpanded forms, (**f** and **g**) dwarfed spores, (**h**) attached, unexpanded spores. Type-II malformations: (**i**) thickened labra with uneven trilete mark, (**j**) thickened and deformed labra and (**k**) thickened labra with growths. Type-III malformations: (**l**) thickened labra with exine cracks. Type-IV malformations: (**m** and **n**) undeveloped trilete mark showing square outline and multiple (quadrilete) marks and (**o** and **p**) oval outline and single (monolete) mark. Type-V malformations: (**q** to **t**, **v** and **w**) specimens with weakly formed trilete marks and/or deformed outline. Type-VI malformations: (**u**) conjoined twin spores

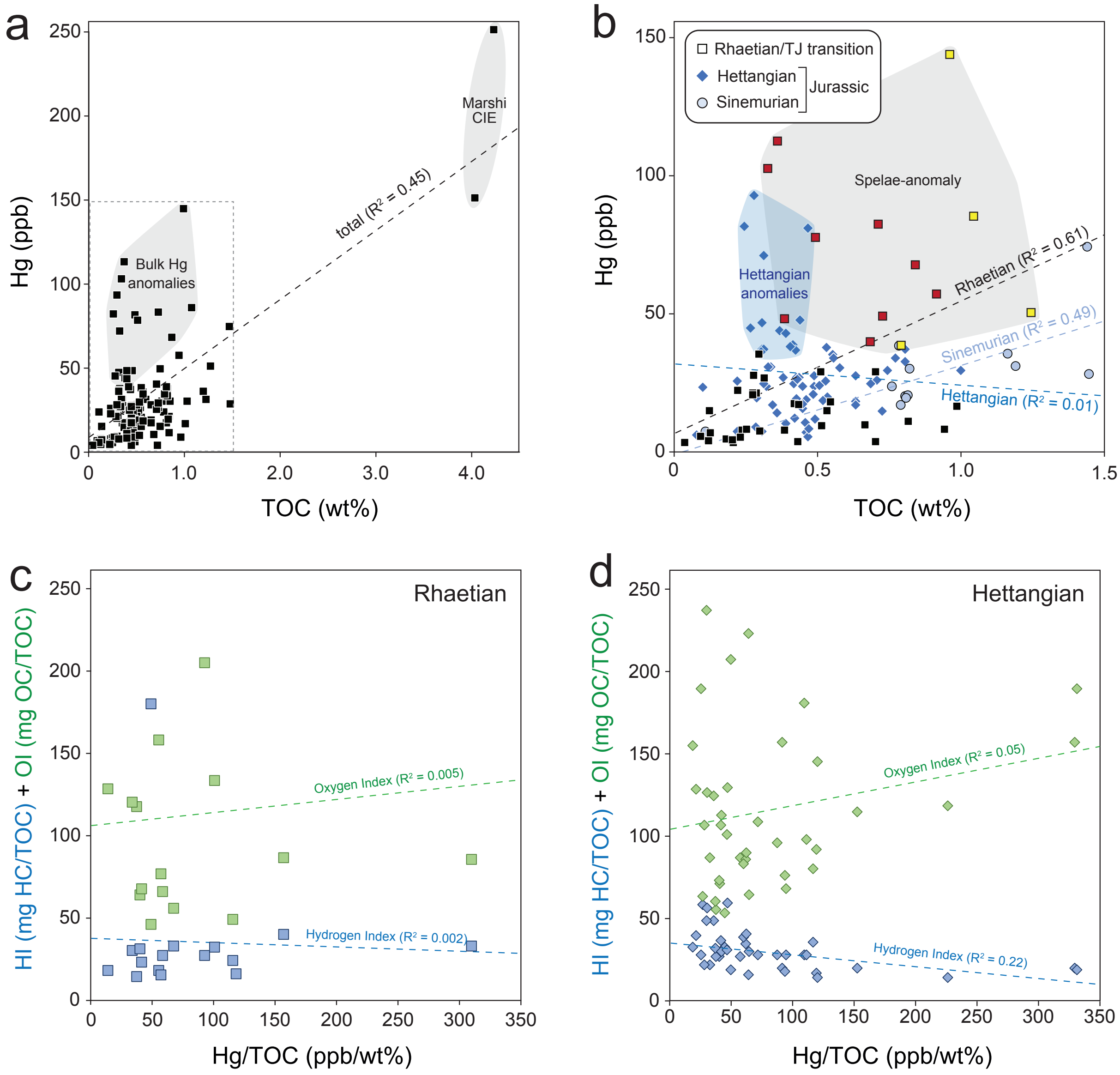

Supplementary Fig. 2. Mercury-organic carbon/matter relations for Schandelah-1 (Lower Saxony Basin). Cross-plots showing linear relation (**a**) total and (**b**) of the different stage Hg relative to TOC. (**a**) The dashed line represents the total linear regression of all the measurements. (**b**) The dashed lines represent the linear regression with the R-squared depicted for the Rhaetian (black squares), Hettangian (blue diamonds) and Sinemurian (light blue circles). The grey shaded area indicates the deviation of the Spelae CIE, and the blue shaded area represents the Hettangian Hg-anomalies. The yellow squares represent increased bulk sedimentary Hg that was likely absorbed and buried through sulphur-drawdown, while the red squares represent volcanic loading. Black squares represent Rhaetian background Hg conditions. (**c** and **d**) The relationship between Hg/TOC values and the Hydrogen Index (blue) and Oxygen Index (green) for the Rhaetian and Hettangian intervals[1].

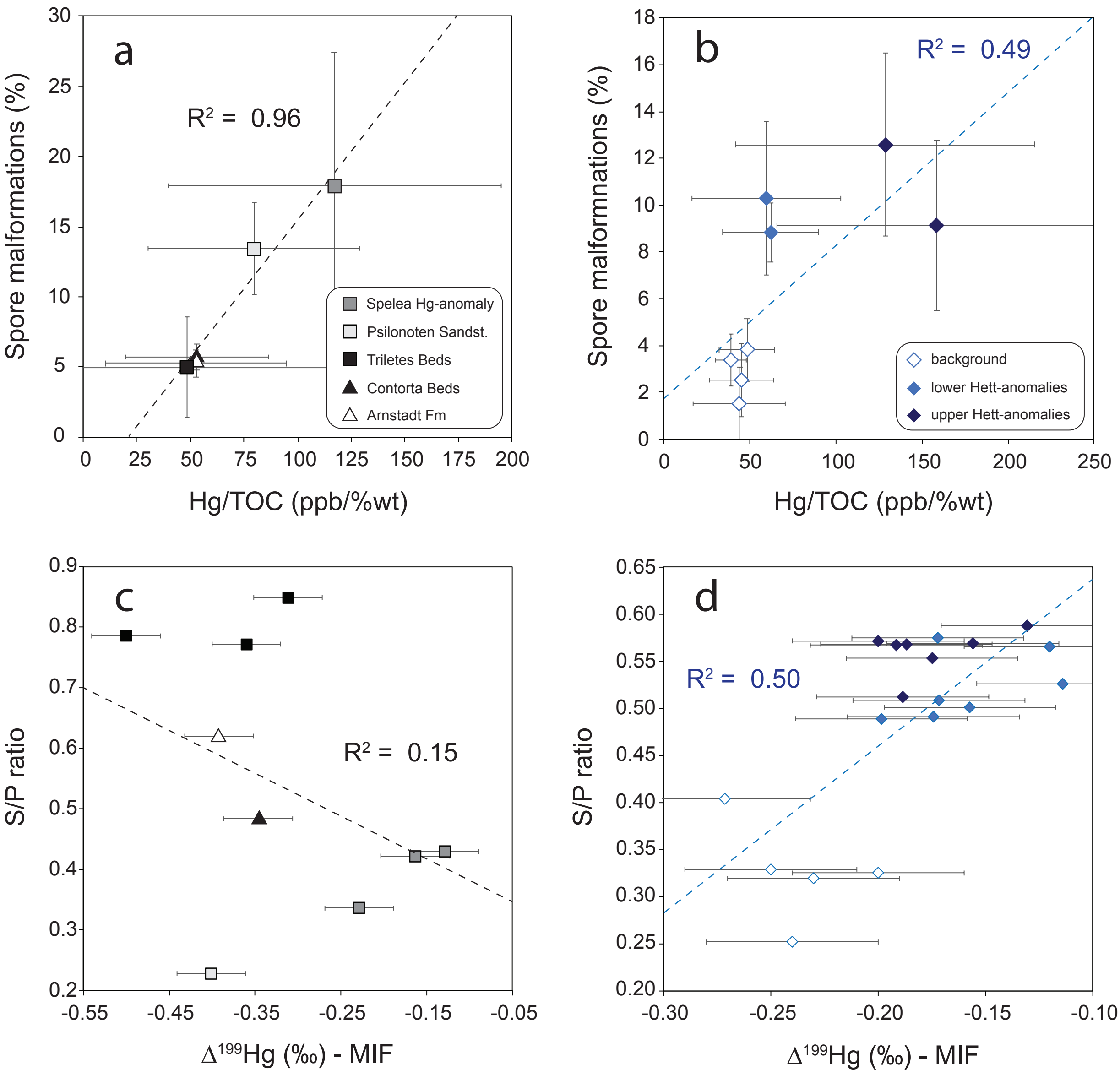

Supplementary Fig. 3. Mercury concentration and isotope relation to vegetation dynamics. Cross-plots of average Hg/TOC concentrations against average spore aberration abundances (**a** and **b**) and Hg-MIF isotope variable (Δ^{199}Hg) against spore/pollen ratio (**c** and **d**) for Triassic-Jurassic transition (grey/black squares) and Hettangian (blue diamonds) intervals. In plots A and B, the average Hg/TOC and spore aberration abundance were calculated for the major lithological intervals in the Rhaetian and lowermost Hettangian (**a**) and for the Hg-enrichment intervals in the Hettangian (**b**). The error bars represent standard deviation (2σ). (**c** and **d**) S/P ratio values were calculated for each mercury isotope measurement, by utilizing the average of the three closed measured values. The error bars indicate the standard deviation (2σ).

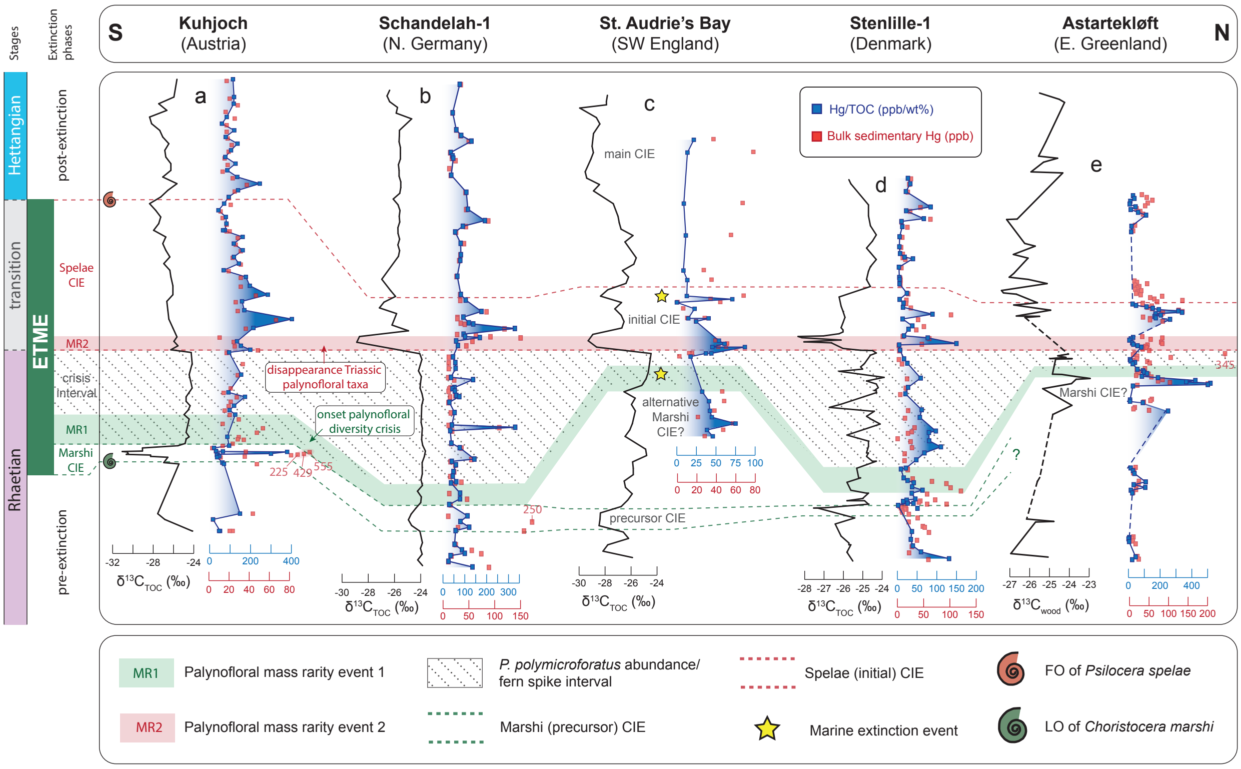

Supplementary Fig. 4. Correlation of δ^{13}Corg records, bulk sedimentary Hg and Hg/TOC concentrations. Correlation of several European records is based on the position of negative carbon isotope excursions and increased extinctions rates as noted in palynofloral assemblages (i.e. mass-rarity 1 and 2 events). The green bar represents mass rarity event 1 and is generally synonymous with the onset of palynofloral diversity crisis. The red bar represents mass rarity event 2 and is equivalent with the disappearance of Upper Triassic palynofloral taxa. In addition, the crisis interval (i.e. *P. polymicroforatus* abundance and/or fern spike interval given in the dotted interval) is utilized to further correlate the position of the end-Triassic Mass-extinction (ETME). This correlation effort is based on previous palynofloral examinations and correlations[2,3]. The position of the Marshi and Spelae CIEs was determined by the last and first occurrences of *Choristoceras marsh*i and *Psiloceras spelae*, respectively, based on the Kuhjoch GSSP section[4]. Increased marine extinction intervals (yellow stars) denote the position of invertebrate extinction[5]. Data is incorporated from (**a**) Kuhjoch (Austria)[4,6,7], (**b**) Schandelah-1 (N. Germany)[1,8], (**c**) St. Audrie's Bay (SW England)[7,9,10], (**d**) Stenlille (Denmark)[2,3,11] and (**e**) Astartekløft (E. Greenland)[7,12,13]. Hg/TOC data from the Kuhjoch and Astartekløft section are replotted by excluding data points with TOC <0.15 wt%, in order to compare these records with the contemporary sites (Schandelah-1, Stenlille-1 and St. Audrie's Bay).

Supplementary References

1 Bos, R. *et al*. Triassic-Jurassic vegetation response to carbon cycle perturbations and climate change. *Global Planet. Change* **228**, 104211 (2023).

2 Lindström, S. Two-phased Mass Rarity and Extinction in Land Plants During the End-Triassic Climate Crisis. *Front. Earth Sci.* **9**, 1079 (2021).

3 Lindström, S. *et al*. A new correlation of Triassic–Jurassic boundary successions in NW Europe, Nevada and Peru, and the Central Atlantic Magmatic Province: A time-line for the end-Triassic mass extinction. *Palaeogeogr. Palaeoclimatol. Palaeoecol.* **478**, 80-102 (2017).

4 von Hillebrandt, A. V. *et al*. The global stratotype sections and point (GSSP) for the base of the Jurassic System at Kuhjoch (Karwendel Mountains, Northern Calcareous Alps, Tyrol, Austria). *Episodes* **36**, 162-198 (2013).

5 Wignall, P. B. & Atkinson, J. W. A two-phase end-Triassic mass extinction. *Earth Sci. Rev.* **208**, 103282 (2020).

6 Bonis, N. R., Kürschner, W. M. & Krystyn, L. A detailed palynological study of the Triassic–Jurassic transition in key sections of the Eiberg Basin (Northern Calcareous Alps, Austria). *Rev. Palaeobot. Palyno.* **156**, 376-400 (2009).

7 Percival, L. M. E. *et al*. Mercury evidence for pulsed volcanism during the end-Triassic mass extinction. *Proc. Natl. Acad. Sci. USA* **114**, 7929-7934 (2017).

8 van de Schootbrugge, B. *et al*. The Schandelah Scientific Drilling Project: A 25-million year record of Early Jurassic palaeo-environmental change from northern Germany. *Newslett. Stratig.* **52**, 249-296 (2019).

9 Bonis, N. R., Ruhl, M. & Kürschner, W. M. Climate change driven black shale deposition during the end-Triassic in the western Tethys. *Palaeogeogr. Palaeoclimatol. Palaeoecol.* **290**, 151-159 (2010).

10 Hesselbo, S. P., Robinson, S. A., Surlyk, F. & Piasecki, S. Terrestrial and marine extinction at the Triassic-Jurassic boundary synchronized with major carbon-cycle perturbation: A link to initiation of massive volcanism? *Geology* **30**, 251-254 (2002).

11 Lindström, S. *et al*. Volcanic mercury and mutagenesis in land plants during the end-Triassic mass extinction. *Sci. Adv.* **5**, eaaw4018 (2019).

12 Mander, L. Taxonomic resolution of the Triassic–Jurassic sporomorph record in East Greenland. J. *Micropalaeontol.* **30**, 107-118 (2011).

13 McElwain, J. C., Wagner, P. J. & Hesselbo, S. P. Fossil plant relative abundances indicate sudden loss of Late Triassic biodiversity in East Greenland. *Science* **324**, 1554-1556 (2009).
