# Supplementary material for: Climate-forced Hg-remobilization associated with fern mutagenesis in the aftermath of the end-Triassic extinction
Source: Nat Commun. 2024 Apr 27;15:3596. doi: 10.1038/s41467-024-47922-0 (PMC11519498; doi:10.1038/s41467-024-47922-0)
Supplement: Supplementary file 3 — Description of Additional Supplementary Information [file 41467_2024_47922_MOESM3_ESM.pdf]

Description of Additional Supplementary Information

Supplementary Data 1: Malformed spore and palynological counts

Supplementary Data 2: Organic isotopes, concentrations and characterization

Supplementary Data 3: Bulk mercury concentrations

Supplementary Data 4: Mercury isotope data

Supplementary Data 5: Mercury isotope standard reference materials recoveries
